# A streamlined implementation of the glutamine synthetase-based protein expression system

**DOI:** 10.1186/1472-6750-13-74

**Published:** 2013-09-24

**Authors:** Rachel Knox, Joanne E Nettleship, Veronica T Chang, Zhao Kun Hui, Ana Mafalda Santos, Nahid Rahman, Ling-Pei Ho, Raymond J Owens, Simon J Davis

**Affiliations:** 1 Radcliffe Department of Medicine and MRC Human Immunology Unit, John Radcliffe Hospital, University of Oxford, Headington, OX3 9DS Oxford, UK; 2 Oxford Protein Production Facility-UK, Research Complex at Harwell, Rutherford Appleton Laboratory, OX11 0FA Oxfordshire, UK; 3 Division of Structural Biology, Henry Wellcome Building for Genomic Medicine, OX3 7BN Oxford, UK; 4 Oxford Centre for Respiratory Medicine, Oxford University Hospital NHS Trust, OX3 7LH, Headington, UK

**Keywords:** Protein expression, Glutamine synthetase, Chinese hamster ovary cells, IRES, HEK 293S

## Abstract

**Background:**

The glutamine synthetase-based protein expression system is widely used in industry and academia for producing recombinant proteins but relies on the cloning of transfected cells, necessitating substantial investments in time and handling. We streamlined the production of protein-producing cultures of Chinese hamster ovary cells using this system by co-expressing green fluorescent protein from an internal ribosomal entry site and selecting for high green fluorescent protein-expressing cells using fluorescence-activated cell sorting.

**Results:**

Whereas other expression systems utilizing green fluorescent protein and fluorescence-activated cell sorting-based selection have relied on two or more sorting steps, we obtained stable expression of a test protein at levels >50% of that of an “average” clone and ~40% that of the “best” clone following a single sorting step. Versus clone-based selection, the principal savings are in the number of handling steps (reduced by a third), handling time (reduced by 70%), and the time needed to produce protein-expressing cultures (reduced by ~3 weeks). Coupling the glutamine synthetase-based expression system with product-independent selection in this way also facilitated the production of a hard-to-assay protein.

**Conclusion:**

Utilizing just a single fluorescence-activated cell sorting-based selection step, the new streamlined implementation of the glutamine synthetase-based protein expression system offers protein yields sufficient for most research purposes, where <10 mg/L of protein expression is often required but relatively large numbers of constructs frequently need to be trialed.

## Background

Mammalian cells are useful for stably expressing recombinant proteins for use in structural and functional studies, as well as for the industrial production of, e.g., therapeutic antibodies and cytokines [1-3]. Mammalian cell-based expression systems are essential when the protein of interest cannot be expressed in bacterial- or yeast-based systems and/or when conventional glycosylation is needed for the folding or stability of the protein (reviewed in [4]). Establishing stable cell lines expressing a given protein typically involves transfection with plasmid vectors carrying the gene of interest and a selection marker [5-7]. Large numbers of resistant clones, often isolated in a multi-well plate format, are then screened to identify high expressers. This process is labor-intensive, time-consuming and limited by the number of clones that can feasibly be screened.

Considerable effort has therefore gone into developing selection strategies requiring reduced screening effort [8-10]. In particular, the development of fluorescence-activated cell sorting (FACS) protocols has significantly increased the throughput of selection using co-expressed fluorescent reporter proteins, *e.g.* green fluorescent protein (GFP), as second selectable markers [11]. Previously, implementation of this approach has involved either two [12] or more (up to five) [13] rounds of FACS selection of the GFP-expressing cells, resulting in these methods still being labor-intensive and taking six months or longer. This effort is justified in the context of the industrial expression of therapeutic proteins, where production can be scaled and repeated indefinitely. For research purposes, however, where milligram quantities of protein may only be required on a one-off basis, faster and less labor-intensive solutions are needed.

We are long-term users of the glutamine synthetase (GS)-based protein expression system, developed by Lonza Biologics, which utilizes a robust viral promoter and selection *via* glutamine metabolism to allow the generation of high-yielding and stable cell lines derived from Chinese hamster ovary (CHO) cells, the major mammalian host for recombinant protein production [6,14]. We previously established cell lines producing ~400 mg/L of a soluble form of the T-cell surface protein, CD4 [6], and yields as high as 5 g/L of antibody have been reported by others in commercial settings [15]. The GS system utilizes the plasmid vector pEE14, which carries the gene of interest and encodes a GS mini-gene. Transfected cells are selected in the presence of graded amounts of the competitive GS inhibitor methionine sulphoximine (MSX), which allows the isolation of cells with very high plasmid copy numbers (>2000/cell [16]). However, CHO cells also readily amplify their own GS gene, necessitating the isolation and screening of single clones, adding 1–2 months to the generation of a high-expressing cell line.

We previously noticed that the expression levels of the top ~50% of protein-expressing clones are generally relatively uniform, which suggested that if weakly expressing clones could be removed along with untransfected resistant cells that had amplified their endogenous GS gene, clone selection might be unnecessary. Here, using both MSX selection and single-step fluorescence-activated cell sorting (FACS) for high co-expression of a green fluorescent protein marker, we establish a streamlined protocol in which cloning is eliminated. With the new method, the transfection-to-protein-purification stages can be completed in just two months. We also show that coupling the GS-based expression system with product-independent selection facilitates the high-level production of hard-to-assay proteins.

## Methods

### Plasmid construction

**Figure 1:**
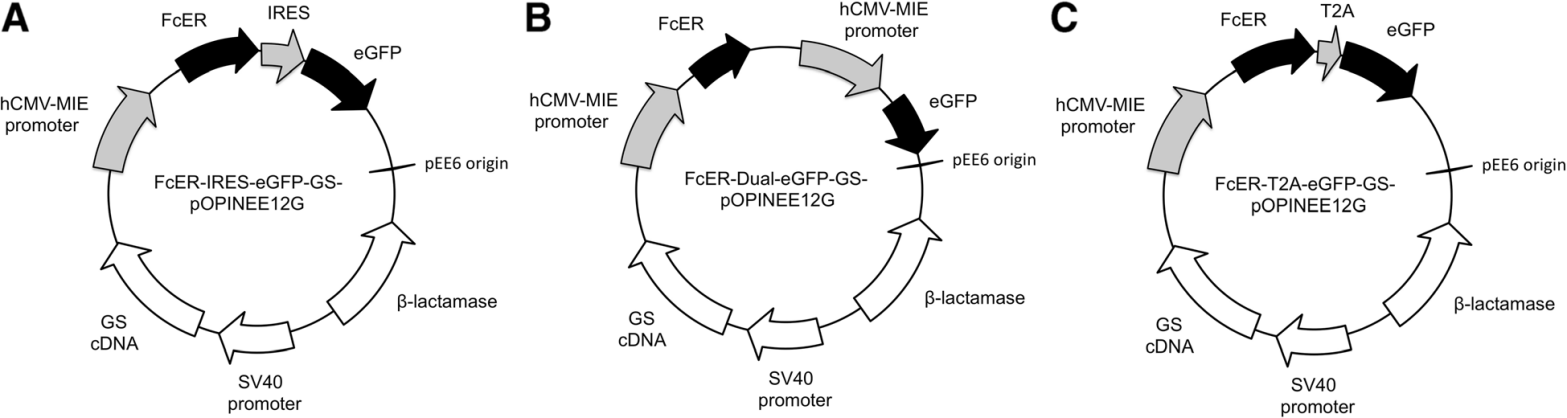
**The principal vectors used in this study.** The three panels show the vector designs for the IRES **(A)**, dual promoter **(B)** and T2A **(C)** versions of FcERα-eGFP-GS-pOPINEE12G (not drawn to scale). The vectors are 9491, 10697 and 8953 base pairs in size, respectively.

The glutamine synthetase vector, pEE12 (Lonza Biologics, Slough, UK) [17], consists of a multiple-cloning site under the control of the human cytomegalovirus (hCMV) promoter, a β-lactamase cassette, and SV40 promoter-driven glutamine synthetase cDNA (GS). The single KpnI site in pEE12 was deleted by site-directed mutagenesis using the Quikchange™ kit (Stratagene, Stockport, UK). A leader sequence and *lacZ* cassette were amplified from the vector pOPING [18] and inserted between the HindIII and EcoRI restriction sites of pEE12 to produce the vector pOPINEE12G (all oligonucleotide sequences are given in Additional file 1: Table S1). IRES-Emerald GFP (eGFP) cDNA was generated by PCR from an existing vector template (pHR-IRES-eGFP [19]) and cloned into the EcoRI/BclI sites of pOPINEE12G. The IgE-specific Fc receptor 1α (FcERα; residues 26–201), the extracellular region of human PD-1 (residues 21–167), or the human chemokine CCL18 (residues 21–89), followed by C-terminal BirA sequence (PD-1 and CCL18 only), hexa-histidine tag and a stop codon, were cloned immediately upstream of this, between the AgeI and EcoRI sites, replacing the *lacZ* gene and creating IRES-eGFP-GS-pOPINEE12G (Figure 1A). An N-terminally tagged version of CCL18 (His-BirA-CCL18) was also generated.

In T2A-eGFP-GS-pOPINEE12G (Figure 1C), expression of the eGFP is linked to the gene of interest *via* a self-cleaving 2A peptide from the insect virus *Thosea asigna* (T2A) [20]. This 18 amino acid sequence was included in the 5’ oligonucleotide used to amplify the eGFP cDNA from pHR-IRES-eGFP. The stop codon between the gene of interest and T2A was omitted. The Dual promoter-GS-pOPINEE12G constructs (Figure 1B) were constructed by cloning the FcERα gene into the KpnI/PmeI sites of pOPINEE12G. eGFP under control of a second hCMV promoter was then added downstream of the FcERα gene by first inserting eGFP into the HindIII/EcoRI sites of pEE6 [21] and then PCR cloning the resulting hCMV promoter–eGFP cassette into the NotI/SalI sites of pOPINEE12G. FcERα was also cloned *via* XbaI into pEE14 [14], the traditional GS system vector.

For making stable FcERα-expressing HEK 293S cells, the GS cDNA in FcERα-IRES-GS-pOPINEE12G was replaced with a bacterial aminoglycoside phosphotransferase 3’ II (*Neo*) gene, which confers resistance to aminoglycoside antibiotics. A transcription unit comprising the *Neo* gene under control of the SV40 early promoter was amplified from pcDNA-DEST40 (Invitrogen, Paisley, UK) and cloned into the NheI/BglII sites of FcERα-IRES-GS-pOPINEE12G creating FcERα-IRES-eGFP-Neo-pOPINEE12G, which enables the selection of transfectants with Geneticin (G418; Sigma Aldrich Company Ltd., Gillingham, UK).

### Cell culture and transfection

CHO-K1 cells were grown at 37°C, 5% CO_2_ in high-glucose Dulbecco's modified Eagle's medium (DMEM; Gibco, Invitrogen) supplemented with 10% fetal calf serum (Sigma Aldrich Company Ltd.), 1% L-glutamine (Sigma Aldrich Company Ltd.), 1% sodium pyruvate (Invitrogen) and an amino acid supplement. For stable transfection, 10^6^ cells (unless otherwise stated) were seeded in a 75 cm^2^ flask. The following day the medium was changed to DMEM supplemented with 10% FCS dialysed against PBS (First Link Ltd., Wolverhampton, UK), 1% sodium pyruvate and amino acids, before the cells were transfected with 10 μg DNA using Genejuice (Novagen, Merck Chemicals Ltd., Hoddesdon, UK) according to the manufacturer’s protocol. The following day, L-methionine sulfoximine (MSX; Sigma Aldrich Company Ltd.) was added to the medium at concentrations ranging from 20–50 μM. Medium was refreshed 5 days after transfection, and again after a further week. Upon the appearance of substantial numbers of clones, cells were removed with Accutase Cell Dissocation Reagent (Gibco, Invitrogen). Cell numbers and viability were assayed by trypan blue exclusion. Cells were spun down and resuspended in PBS, ready for FACS. The FACS-sorted cells were then expanded in MSX-containing medium as above. Once cells were confluent in cell factories or final-stage 175 cm^2^ flasks, sodium butyrate was added at a concentration of 2 mM.

HEK 293S cells were grown in high-glucose DMEM supplemented with 10% FCS and 1% L-glutamine. Transfection was carried out as above, using G418 disulphide salt (Invitrogen) at a concentration of 0.8 mg/mL to select for Geneticin-resistant clones.

### Flow cytometry and preparative FACS

GFP expression of transfected CHO and HEK 293S cells was monitored via flow cytometry on a CyAn ADP Analyzer (Beckman Coulter, Krefeld, Germany). Preparative FACS was performed on a MoFlo high-speed cell sorter (Beckman Coulter). The argon-ion laser was tuned to 488 nm with 100 mW of power, and eGFP fluorescence detected in FL1 through a 530/40-nm bandpass filter. The top 30% (unless otherwise stated) of live eGFP-expressing cells were sorted into a single tube, stored on ice. Data analysis was performed using FlowJo software (Tree Star Inc., Ashland, OR, USA).

### ELISA

FcERα receptor yields were determined by competition ELISA. Supernatant was sampled from confluent 175 cm^2^ flask cultures of FACS-sorted cells, three weeks after the addition of sodium butyrate. ELISA plates (Costar, Corning Incorportated, New York) were coated with 50 μL purified FcERα at 10 μg/mL and incubated at 4°C overnight. The next day, this was removed and the plate washed three times with PBS 0.05% Tween 20 (Sigma Aldrich Company Ltd.), before blocking with 100 μL PBS 1% casein (VWR, Lutterworth, UK) for 30 minutes at room temperature. Meanwhile, competition mixtures consisting of 55 μL of serially titrated sample or standard (purified FcERα), and 55 μL mouse anti-FcERα antibody (AbCam, Cambridge, UK) at 3.3 mg/L were prepared, and incubated at 37°C for 30 minutes. After washing the plate as before, 50 μL competition mixture was then added to each well, and the plate was incubated at 4°C for 1 hour. After another wash, 50 μL hydrogen peroxidase-coupled goat anti-mouse IgG Fc (Sigma Aldrich Company Ltd., diluted 1 in 2000 in DMEM) was added, and the plate incubated at 4°C for a further hour. Peroxidase detection was *via* TMB substrate (Thermo Scientific, Hemel Hempstead, UK) according to the manufacturer’s protocol. FcERα titre was determined by plotting the absorbance of titrated samples, and reading off the dilution factor at 50% inhibition, compared to the standard. Where necessary the PD-1 yield was similarly determined by competition ELISA, using mouse anti-human-PD-1 antibody clone 2 (unpublished data, S. Morgan *et. al.*) at 2.5 mg/L, and HRP-coupled anti-mouse IgG Fc as above.

For identifying peak fractions containing CCL18, the sandwich ELISA method embodied in the Human CCL18/PARC Quantikine ELISA kit (R&D systems, Inc., Minneapolis) was employed. The kit was used according to the manufacturer’s instructions.

### Purification with Ni-NTA column

The soluble His-tagged proteins were purified from the supernatant on a Ni-NTA column followed by gel filtration as previously described [22].

## Results and discussion

### Optimization of FACS-based selection

We set out to establish a shortened protocol for generating stable CHO cell cultures expressing proteins of interest using the glutamine synthetase-based gene expression system. Our principal test protein was the Type I IgE-specific Fc receptor (FcERα), which is involved in the control of allergic responses [23,24]. We compared three approaches for co-expressing the eGFP selectable marker (see below), but initial optimization of the method was based on a vector that expressed eGFP from an internal ribosome entry site (IRES). The gene encoding a soluble form of FcERα (residues 26–201) [25] was cloned into the pOPINEE12G expression vector downstream of a start codon and sequence encoding a heterologous signal peptide, and upstream of sequence encoding an IRES and Emerald GFP (eGFP) reporter (residues 1–240), giving FcERα-IRES-eGFP-GS-pOPINEE12G (Figure 1A). Expression of both genes is in this way controlled by the human cytomegalovirus promoter, with the downstream position of the eGFP reporter ensuring that its expression will be lower than that of FcERα. Following transfection of CHO-K1 cells in a 75 cm^2^ flask with this vector, the cells were left undisturbed for 3 weeks, after which substantial numbers of clones were visible by eye. In preliminary experiments using 40 μM MSX, a low seeding density of 10^6^ cells/flask produced the largest numbers of resistant clones (>200) (Additional file 2: Figure S1); cells at higher initial densities overgrew in the first week when MSX selection was presumably beginning to take effect.

**Figure 2:**
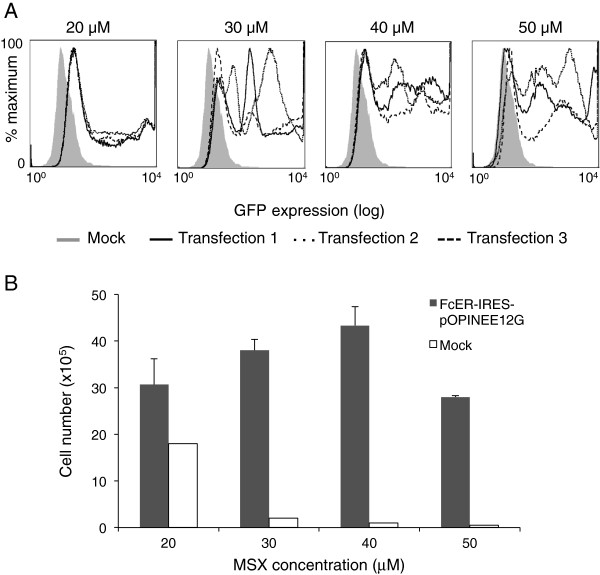
**Cell yields and eGFP expression prior to cell sorting. A**. eGFP expression by cells from triplicate transfections selected at MSX concentrations of 20-50 μM. Populations were gated on live cells. **B**. Cell recovery on the day of cell sorting. Cell growth and eGFP expression were best at 40 μM MSX. Error bars indicate standard errors for triplicate transfections.

**Figure 3:**
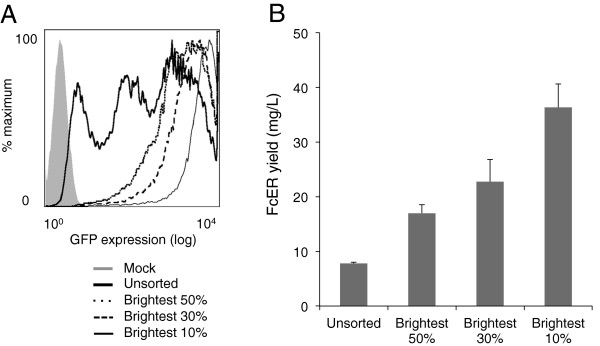
**FcERα yields following cell sorting for different levels of eGFP expression.** Triplicate transfections were performed and clones selected at 40 μM MSX. After three weeks, cells were removed with Accutase and sorted for the brightest 50%, 30% or 10% of the population. An unsorted population was also maintained. Flow cytometry was performed on these populations a week after the sort (**A**, gated on live cells), prior to populations being used to seed 175 cm^2^ flasks, which were treated with sodium butyrate upon confluency and left for three weeks before supernatant samples were collected. FcERα yield, determined by competition ELISA, shows the advantages of sorting for high levels of expression **(B)**.

Our initial goal was to optimize expression versus cell recovery since high MSX concentrations were expected to give higher expression but fewer clones, slowing the production of protein-expressing cultures. Selection at a range of MSX concentrations, from 20 to 50 μM showed that eGFP expression at 20 μM was uniformly low, with transfection variability increasing at higher MSX concentrations (Figure 2A). Cell recoveries and eGFP expression were optimal after selection at 40 μM (Figure 2A,B). Transfected cells (in 3 × 75 cm^2^ flasks) selected at this MSX concentration were pooled and sorted for the brightest 10%, 30% or 50% of cells by FACS; the results of one such sort are shown in Figure 3A. Each sorting run yielded >10^5^ cells, which grew to confluence in a 175 cm^2^ flask within a week. Sodium butyrate was added to enhance protein expression [26], and after three weeks FcERα yields were determined by competition ELISA (Figure 3B). The advantages of FACS-based selection are immediately apparent: sorting of the brightest 10% of cells yields five-fold more FcERα than the unsorted population, and nearly double that of the brightest 30%. To maximize cell recovery and to speed line generation, however, lines established from the top 30% of expressers were selected for further characterization.

**Figure 4:**
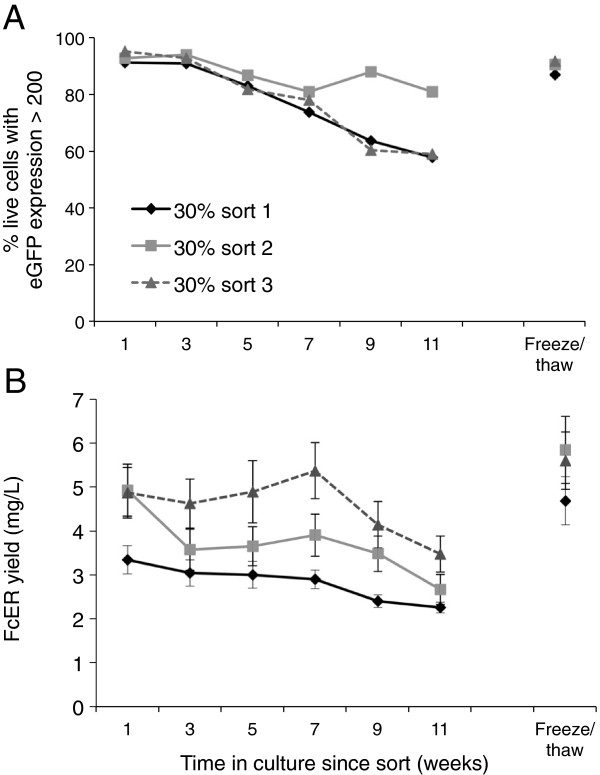
**Stability of the sorted cell populations.** eGFP expression (**A**, gated on live cells) and FcERα yield **(B)** of FcERα-IRES-eGFP-GS-pOPINEE12G transfected CHO cells are stable for the first month after sorting, but then decrease over time. Cells were passaged in 75 cm^2^ flasks, split twice weekly. Every two weeks, 4×10^6^ cells were used to seed a 75 cm^2^ flask. Forty-eight hours later, supernatants were collected for FcERα titre determination by competition ELISA, and for testing eGFP expression by flow cytometry. The levels of expression are lower than those in Figure 3 owing to the shorter period for accumulation of the protein. Error bars indicate standard errors for triplicate ELISA readings. “Freeze/thaw” denotes cells frozen at week 1 and thawed at week 11.

The stability of the sorted cell populations was investigated during continuous culture for a period of three months. Flow cytometry, performed every two weeks, showed that eGFP expression decreases by up to 40% with time, presumably as high-expressing cells are out-grown by low-expressing cells not excluded by cell-sorting (Figure 4A). A similar loss of FcERα expression was also observed over this period (Figure 4B). Importantly, however, both eGFP and FcERα expression levels are constant for the first month, the maximum period for which large-scale cultures would likely be kept. Cells can always be re-sorted for eGFP expression if expression levels drop significantly. eGFP expression is very stable upon freeze-thawing of the cells (Figure 4A,B).

### Comparison of methods for co-expression of eGFP

**Figure 5:**
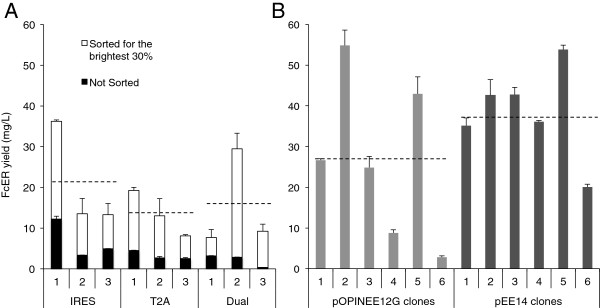
**Comparison of the effectiveness of the IRES, dual promoter and T2A versions of the FcERα-eGFP-GS-pOPINEE12G vector for expressing FcERα in sorted cell populations, versus the yields obtainable following clone selection. A**. FcERα expression from stable pOPINEE12G transfected cells was variable between triplicate transfections, with the IRES version giving slightly, but not significantly higher FcERα yields. Sorting for the brightest 30% considerably improved yield compared to unsorted populations of the same cells. **B**. FcERα yield from clonal cell lines obtained by conventional limiting dilution cloning. Clones 1 and 2 were selected at 35 μM MSX; clones 3-6 at 40 μM. FcERα titre was determined by competition ELISA on supernatant samples collected from 175 cm^2^ flasks after three weeks. Average yields from the triplicate sorted cell populations are indicated by the dotted lines; error bars indicate standard errors for triplicate ELISA readings.

The efficiency with which eGFP-based FACS allows selection of cells expressing the highest levels of the protein of interest depends on the relationship between the expression levels of the two proteins. In an effort to vary this relationship, two other co-expression strategies were trialed. Firstly, a version of the pOPINEE12G vector was generated wherein eGFP expression was driven by a second CMV promoter (referred to as the “dual promoter” vector, FcERα-Dual-eGFP-GS-pOPINEE12G; Figure 1B). Secondly, the 18 amino acid self-cleaving 2A peptide from the insect virus *Thosea asigna* (T2A) [20] was used to link the translation of the protein of interest directly to that of eGFP, in the vector FcERα-T2A-eGFP-GS-pOPINEE12G (Figure 1C). Three weeks after transfection with each vector, half of the cells in each flask were sorted by FACS for 30% of cells expressing the highest eGFP levels; the remainder was left unsorted. Both sets of cells were expanded into 175 cm^2^ flasks, treated with sodium butyrate and left for three weeks. Competition ELISAs once again revealed significant variation between triplicate transfections performed on the same day (Figure 5A). Overall, the IRES-based vector gave slightly higher average FcERα expression, although the differences were not significant (p > 0.05), owing to the considerable variability in FcERα expression by the FACS-sorted cells. The high level of variability was unexpected and possibly arises from the amplification of small differences in the transfected cultures present at the time of sorting. It suggests that the protocol would benefit from pooling three or more transfected flasks of cells prior to sorting. A second test protein, *i.e.* a soluble form of human PD-1, a transmembrane protein that regulates T- and B-cell responses [27], gave similar results (Additional file 3: Figure S2).

### Productivity of FACS-selected lines versus clones

The question arises of how the yields from the FACS-selected lines compare with those of clones generated with FcERα-IRES-eGFP-GS-pOPINEE12G. We also wanted to make comparisons with clones obtained using pEE14, the traditional vector used with CHO cells, which utilizes a GS mini-gene [14] rather than a GS cDNA. The FcERα gene was cloned into pEE14 and stable clones generated with this vector and FcERα-IRES-eGFP-GS-pOPINEE12G. The six best-expressing clones in each case, based on initial dot-blot analysis as described in [6], were expanded to 175 cm^2^ flask cultures, treated with sodium butyrate and left for 3 weeks. The average expression of the pEE14-derived but not the pOPINEE12G-derived clones was significantly higher (p = 0.002) than that of the FACS-selected lines, but this amounted to a less than 1.7-fold difference in expression (Figure 5B). Similarly, the best of the clones expressed the protein only ~1.6 fold better than the best of the sorted lines. However, the most important comparison is between the best of the clones and the average expression of the FACS-selected lines, assuming that multiple transfections will be pooled prior to sorting in order to reduce the effects of transfection variability. In this case the best clone performs 2.5-fold better than the pOPINEE12G-derived FACS-selected line (taking the average of the three replicates). It needs to be borne in mind, however, that if needed, better expressing lines could be obtained by FACS-selecting the top 10% of expressers. It is possible that the synthesis of an additional protein (eGFP) may burden the cell machinery in the case of the FcERα-IRES-eGFP-GS-pOPINEE12G transfected clones and lines, accounting for the reduced expression [13].

### Utility of the method in other selection systems

**Figure 6:**
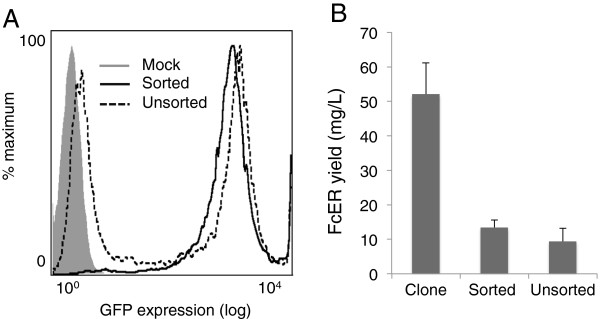
**FcERα expression by stably transfected HEK 293S cells. (A)**. eGFP expression by sorted and unsorted FcERα-IRES-eGFP-Neo-pOPINEE12G transfected cells, one week after cell sorting. FcERα yield from these cells was four-fold lower than that of a clonal cell line (Neo clone 32), isolated previously by conventional limited dilution cloning. FcERα titre was determined by competition ELISA on supernatant samples collected from 175 cm^2^ flasks after three weeks **(B)**. Error bars indicate standard errors for triplicate ELISA readings.

We also sought to determine whether single-step FACS-based selection for a fluorescent marker would be useful in the context of other types of selection. The bacterial aminoglycoside phosphotransferase 3’ II (*Neo*) gene, which confers resistance to aminoglycoside antibiotics, including Geneticin (G418 Sulfate), is widely used to select transfected mammalian cells [28]. This enables the survival of any cell bearing one copy of the gene, whereas for the GS system, only cells with very high plasmid copy numbers survive selection. We transferred the *Neo* gene to the pOPINEE12G vector, generating FcERα-IRES-eGFP-Neo-pOPINEE12G, and used it to transfect embryonic kidney (HEK) 293S cells lacking N-acetylglucosaminyltransferase I (GnTI) activity, which are used for the expression of recombinant proteins devoid of complex N-glycans [29]. A high concentration of G418 (0.8 mg/mL) allowed the selection of uniformly high eGFP-expressing cells (Figure 6A), from which it was difficult to select a high-expressing fraction using FACS. Sorting for the single population of eGFP-expressing cells yielded a line expressing 13 mg/L of FcERα (Figure 6B). However, in contrast to CHO cells selected with MSX, there was little, if any, advantage obtained by sorting, whereas up to ~4-fold higher expression was obtainable by cloning the cells (Figure 6B).

### An example: CCL18

**Figure 7:**
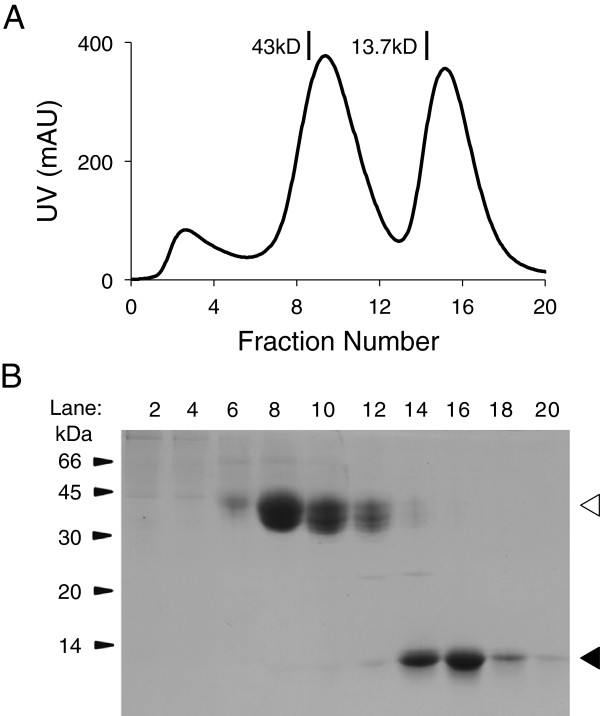
**Expression of CCL18. A**. Elution profile for Ni-NTA-purified CCL18 (second peak) eluting from a Superdex 75 HR 10/30 gel-filtration column. Peak 1 corresponds to an unidentified histidine-containing impurity detectable with anti-pentahistidine antibodies on Western blots (data not shown). Fractions containing this protein lacked CCL18 reactivity according to a sandwich ELISA assay (data not shown). The elution positions of two proteins of known size (43 and 13.7 kDa) are indicated. **B**. SDS-PAGE analysis of the proteins eluting from the G-75 column under non-reducing conditions. The identity of the small protein in the late-eluting peak (black arrow) was confirmed as CCL18 using the ELISA assay (data not shown). The identity of the ~40 kDa protein (white arrow) was not determined.

The identification of the highest-expressing clones using the GS system conventionally relies on there being a convenient small-scale assay for the target protein soon after the clones appear. However, the target-independent nature of FACS-based selection and close-to-maximum level of expression thus obtainable (*i.e.* within a factor of 2–2.5 fold of the best clone) obviates the need for any small-scale or intermediate assays, which is particularly helpful when the protein target is difficult to assay. Illustrating this, we obtained high-level expression of a biotinylatable form of CCL18, a 7.8 kDa orphan chemokine implicated in the lung-specific recruitment of lymphocytes to, *e.g.*, the lung in asthmatics [30], using the new approach. It was not clear whether folding of the chemokine would tolerate the N- or C-terminal addition of biotinylation sequences, so constructs tagged at either end were tried. In our experience relatively small proteins cannot be easily transferred to nitrocellulose for Western blotting, which is among the simplest ways to assay for expression, and this was the case for CCL18 (data not shown). We therefore eschewed assaying for CCL18 at early stages of production and instead waited until we could determine how much Ni-NTA-reactive protein could be purified from 2L cultures grown from FACS-selected eGFP-expressing cells, prepared as described above. The C-terminally tagged protein failed to express at all (data not shown), whereas the yield of well-folded N-terminally tagged CCL18 was approximately 3 mg/L (Figure 7).

## Conclusions

**Figure 8:**
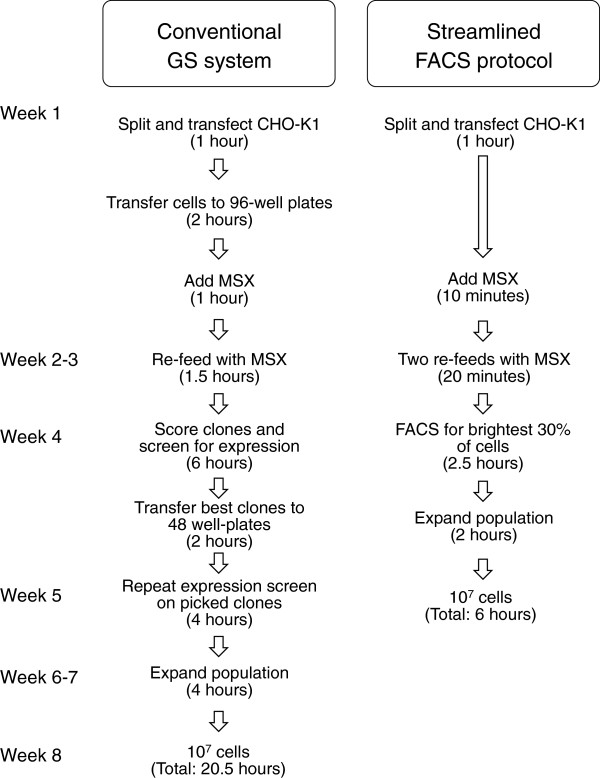
**Comparison of conventional GS system-based, stable cell-line isolation using limiting dilution cloning, with the new protocol.** Labor hours include media preparation and are based on, in the case of the conventional, cloning-based method (left), the production of 10^7^ clonal cells, for which nine 96-well plates are plated out in addition to those needed for mock transfections. For the new protocol (right), the calculation of labor hours is based on the generation of a starting population of 10^7^ cells obtained following the flow-cytometric sorting of three transfected 75 cm^2^ flasks of cells.

The expression of recombinant mammalian proteins is time-consuming and labor-intensive. We have established a streamlined method for implementing the glutamine synthetase-based protein expression system, based on the co-expression of GFP driven by an IRES sequence alongside the gene of interest. The new FACS-based selection protocol is compared with the conventional, cloning-based approach in Figure 8. The principal savings are in the form of handling steps (reduced by almost half) and handling time (reduced by ~75%). However, the method also reduces, by ~ 3 weeks, the time needed to produce a protein-secreting culture of 10^7^ cells. This estimate is based on FACS selection of 3 × 75 cm^2^ flasks of cells (i.e. ~1.2 × 10^7^ cells) from which 10^6^ cells are recovered, and could be further reduced by sorting larger numbers of cells. Overall, the new approach yielded cell populations that expressed our protein of interest, FcERα, relatively stably over a period of 5 weeks, at levels >50% of that obtainable with an “average” clone, and within ~40% of the best clone. No significant advantages were obtained by using dual promoters to express the two proteins, or 2A sequences. The success of the new approach likely hinges on the inherent strength of the expression system since previous attempts to utilize fluorescence-based selection used two [12] or more (up to 5) [13] rounds of FACS selection. The ease with which close-to-optimal expression is obtainable in a single step with the new approach, coupled with the target protein-independent selection of expressing cells, offsets some of the risk in producing hard-to-assay proteins, as exemplified by our expression of biotinylatable CCL18.

In using the new method the trade-off between speed and expression levels could nevertheless be further considered. When low-expressing proteins are being studied, higher percentage sorts may be suitable, and in extreme cases, single-cell sorting could be applied to generate a highly expressing clone. One slight drawback with the new method is its dependence on the efficiency of transfection, which we generally find to be variable on a flask-to-flask basis over extended culture periods. However, combining replicate transfected flasks easily circumvents this issue. A final matter is that sorting for high expression is only worthwhile when there is large variability of expression among the selected cells. In the case of G418 selection, for which the levels of expression among GFP-positive cells was very uniform, almost no advantage was gained by sorting the GFP expressing cells, whereas substantially increased expression was obtainable by conventional cloning. Although unsuitable, of course, for prolonged expression of therapeutic proteins on an industrial scale, this method provides yields more than sufficient for research purposes, where less than 10 mg/L of protein is generally required and often large numbers of constructs have to be trialed.

## Competing interests

The authors declare that they have no competing interests.

## Authors’ contributions

RK, RJO and VTC generated the expression constructs and the sorted-cell populations. RK, ZKH, AMS and NR participated in the protein purification, and RK and JEN undertook expressed-protein detection and identification. SJD, RJO and L-PH conceived the study. RK, RJO and SJD drafted the manuscript. All authors read and approved the final manuscript.

## Supplementary Material

Additional file 1: Table S1 Primer sequences used for generating the pOPINEE12G expression vectors. Restriction sites are underlined; infusion tag sequences are indicated in parentheses. F = forward primer; R = reverse primer. Click here for file

Additional file 2: Figure S1 Clone growth versus seeding density. Cell recovery (*i.e.* clone growth), determined three weeks after transfection, was highest following seeding at low initial densities of 10^6^ cells/flask. Click here for file

Additional file 3: Figure S2 Soluble PD-1 expression from IRES, T2A and dual promoter pOPINEE12G-stably transfected sorted or unsorted CHO-K1 cells. PD-1 titre was determined by competition ELISA on supernatant samples collected from 175 cm^2^ flasks after three weeks. As with FcERα, results between triplicate transfections were variable, with the IRES-containing vector giving on average slightly higher PD-1 yields. Average yields from the triplicate sorted cells are indicated by the dotted lines; error bars indicate standard errors for triplicate ELISA measurements. Click here for file
