# Supplementary material for: A streamlined implementation of the glutamine synthetase-based protein expression system
Source: BMC Biotechnol. 2013 Sep 24;13:74. doi: 10.1186/1472-6750-13-74 (PMC3850363; doi:10.1186/1472-6750-13-74)
Supplement: Additional file 1: Table S1 — Primer sequences used for generating the pOPINEE12G expression vectors. Restriction sites are underlined; infusion tag sequences are indicated in parentheses. F = forward primer; R = reverse primer. [file 1472-6750-13-74-S1.doc]

**SUPPLEMENTARY TABLE**

**Table S1.** Primer sequences used for cloning the pOPINEE12G expression vectors. Restriction sites are underlined; infusion tag sequences are indicated in parentheses. F = forward primer; R = reverse primer.

|  | F/R (restriction sites) | Primer sequence 5’ to 3’ |
| --- | --- | --- |
| KpnI deletion from pEE12 *via* mutagenesis | F | CAAGCGGCACCGCTACCACATTCGAG |
|  | R | ctcgaatgtggtagcggtgccgcttg |
| μ-phosphatase leader-*LacZ* | F (HindIII silenced) | (CCTTGACACGAAGCT)GCCACCATGGGGATCCTTCCCAGCC |
|  | R (EcoRI) | (TATGATCAATGAATT)CTTAGTGATGGTGATGGTGATG |
| IRES-eGFP | F (EcoRI) | TAGTAGGAATTCCGCCCCTCTCCCTCCCCCCCCCCT |
|  | R (BglII) | CTACTATGATCATTACTTGTACAGCTCGTCCATGCCGAG |
| FcERα-His | F (AgeI) | TAGTAGACCGGTGTCCCTCAGAAACCTAAGGTCTCC |
|  | R for IRES- pOPINEE12G^a^ (EcoRI) | CTACTAGAATTCTTAGTGATGGTGATGGTGATGTTT |
|  | R for T2A-pOPINEE12G^a^ (EcoRI) | CTACTAGAATTCGTGATGGTGATGGTGATGTTTCTTCTC |
| PD-1-BirA-His | F (AgeI) | TAGTAGACCGGTCCAGGATGGTTCTTAGACTCCCCAGA |
|  | R (BirA, MscI^b^) | CTACTATGGCCACTCGATTTTCTGTGCCTCGAAGATGTCATTCAGCCCGGGTTGGAACTGGCCGGCTGACCTGGT |
| T2A-eGFP | F (EcoRI) | TAGTAGGAATTCGAGGGCAGAGGAAGTCTTCTAACATGCGGTGACGTGGAGGAGAATCCTGGGCCCATGGTGAGCAAGGGCGAGGAGC |
| CCL18-BirA-His^c^ | F (AgeI, CCL18 signal peptide) | TAGTAGACCGGTGCCACCATGAAGGGCCTTGCAGCTGCCCT |
|  | R (BirA, His, EcoRI) | CTACTAGAATTCTTAGTGATGGTGATGGTGATGTTTCCACTCGATTTTCTGTGCCTCGAAGATGTCATTCAGGGCATTCAGCTTCAGGTCGCTGAT |
| His-BirA-CCL18 | F (AgeI, His, BirA, CCL18 mature protein) | TAGTAGACCGGTAAACATCACCATCACCATCACCTGAATGACATCTTCGAGGCACAGAAAATCGAGTGGGCACAAGTTGGTACCAACAAAGAGCT |
|  | R (EcoRI) | CTACTAGAATTCTTAGGCATTCAGCTTCAGGTCGCTGAT |
| FcERα for dual promoter constructs | F | (GCGTAGCTGAAACCG)GCGTCCCTCAGAAACCTAAGGTCT |
|  | R | (GTGATGGTGATGTTT)CTTCTCACGCGGAGCTTTTATT |
| eGFP for pEE6 | F (HindIII silenced) | (CCTTGACACGAAGCT)ATGGTGAGCAAGGGCGAGG |
|  | R (EcoRI silenced) | (TATGATCAATGAATT)TTACTTGTACAGCTCGTCCATC |
| hCMV-eGFP for pOPINEE12G | F (NotI) | (tcatgtctggcggcc)gccgatatttgaaaatatg |
|  | R (SalI) | (gcccgaggtcgacgc)tctcccttatgcgactc |
| FcERα-His for pEE14 | F^d^ (XbaI) | TAGTAGTCTAGAGAAGCTGCCACCATGGGGATCCTT |
|  | R (XbaI) | CTACTATCTAGATTAGTGATGGTGATGGTGATGTTTCTTCTC |
| Neo | F (BglII) | (TTTGCAAAAAGCTAGC)CTGTGGAATGTGTGTCAGTTAG |
|  | R (NheI) | (TCCTTCACAAAGATCT)TAGCTAGAGGTCGACGGTATC |

^a^ For IRES versions, a stop codon is required at the 3’ end of the His tag. In T2A vectors, this stop codon is omitted to allow continuous translation into the eGFP *via* the skip sequence.

^b^ PD-1-BirA was inserted via blunt end ligation upstream of the His tag in FcERα-IRES-eGFP-GS-pOPINEE12G and FcERα-T2A-eGFP-GS-pOPINEE12G.

^c^ The native CCL18 signal peptide was used here. The PCR product was cloned into a version of IRES-eGFP-pOPINEE12G in which the AgeI site was moved 200 base pairs upstream, *i.e.* beyond the signal peptide generally used, in order to allow native signal sequences to be used.

^d^ FcERα was PCRed from FcERα-IRES-eGFP-GS-pOPINEE12G, from the signal peptide region, as pEE14 does not have an intrinsic signal peptide sequence.
