# Supplementary figures and images for: A streamlined implementation of the glutamine synthetase-based protein expression system
Source: BMC Biotechnol. 2013 Sep 24;13:74. doi: 10.1186/1472-6750-13-74 (PMC3850363; doi:10.1186/1472-6750-13-74)

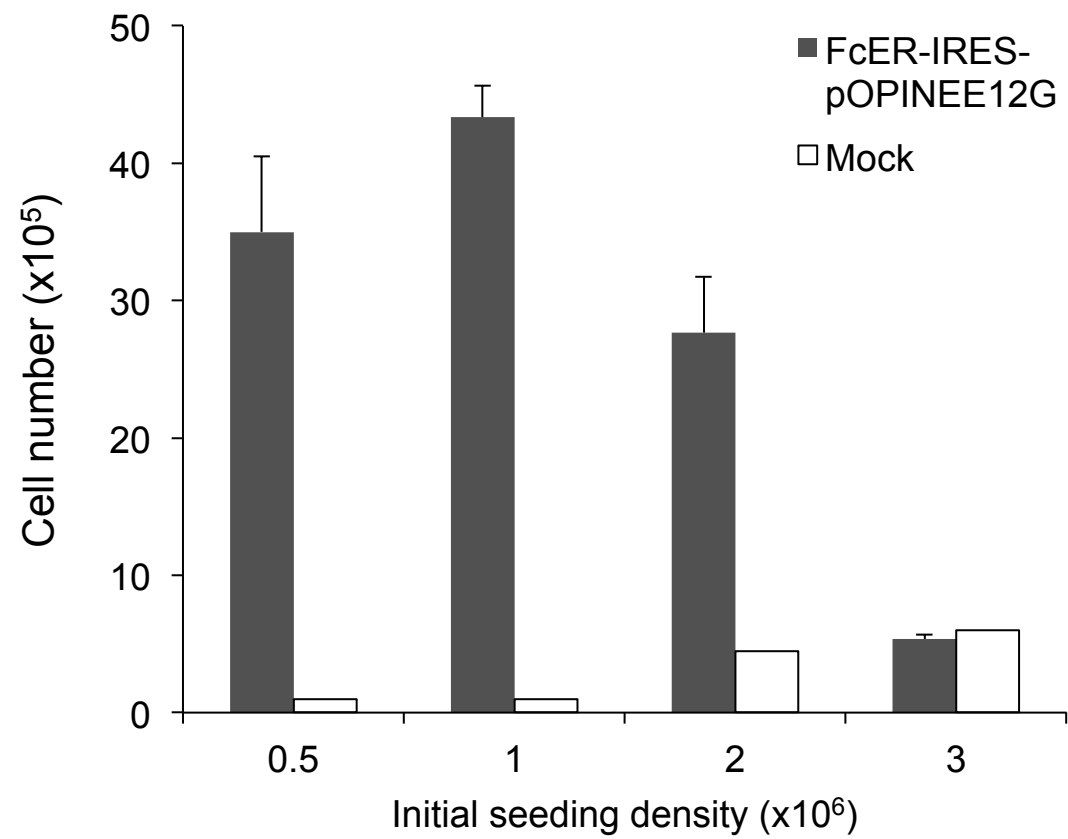

Supplement: Additional file 2: Figure S1 — Clone growth versus seeding density. Cell recovery (i.e. clone growth), determined three weeks after transfection, was highest following seeding at low initial densities of 106 cells/flask. [file 1472-6750-13-74-S2.pdf]

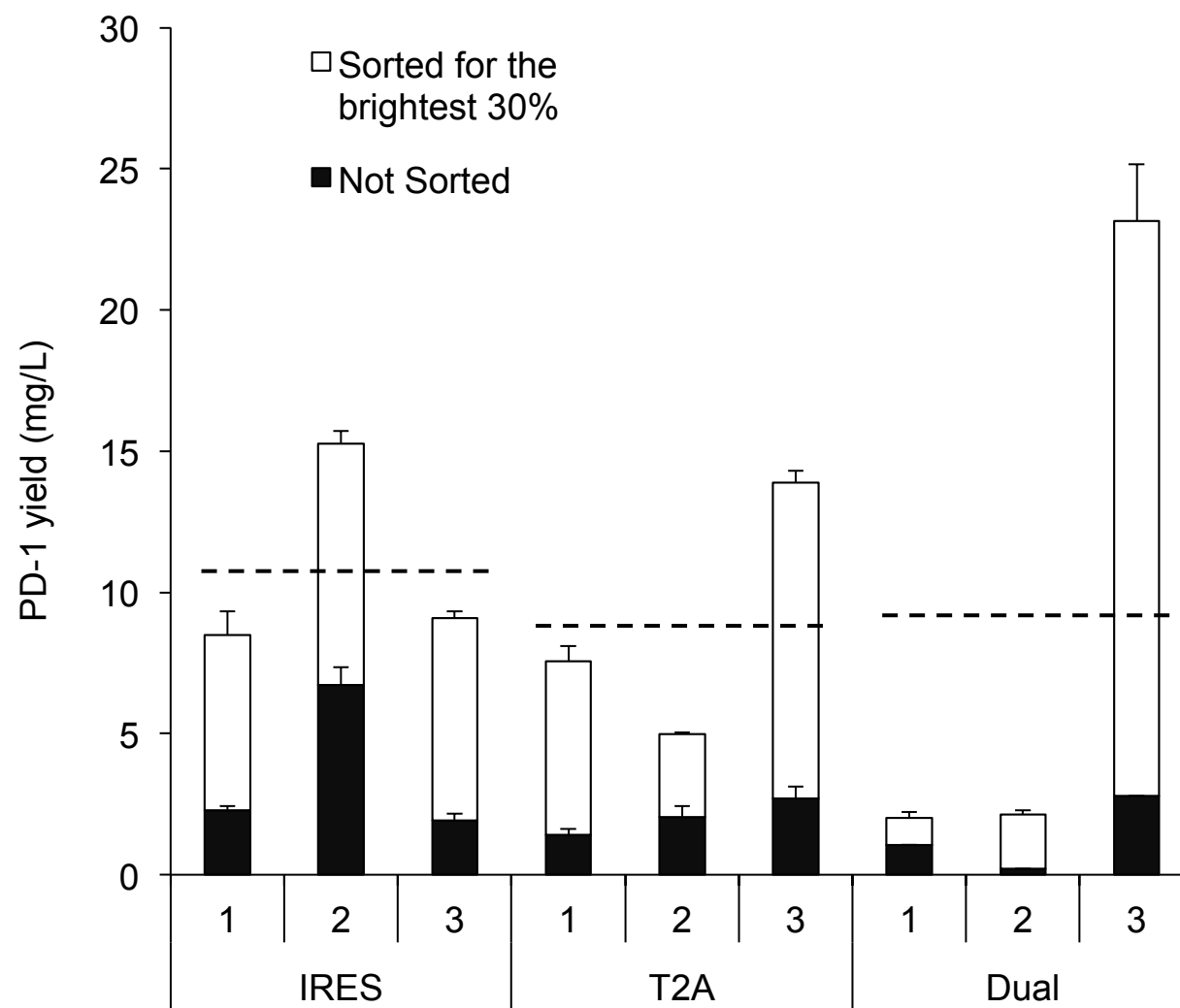

Supplement: Additional file 3: Figure S2 — Soluble PD-1 expression from IRES, T2A and dual promoter pOPINEE12G-stably transfected sorted or unsorted CHO-K1 cells. PD-1 titre was determined by competition ELISA on supernatant samples collected from 175 cm2 flasks after three weeks. As with FcERα, results between triplicate transfections were variable, with the IRES-containing vector giving on average slightly higher PD-1 yields. Average yields from the triplicate sorted cells are indicated by the dotted lines; error bars indicate standard errors for triplicate ELISA measurements. [file 1472-6750-13-74-S3.pdf]
